# Evaluation of animal model congruence to human depression based on large-scale gene expression patterns of the CNS

**DOI:** 10.1038/s41598-021-04020-1

**Published:** 2022-01-07

**Authors:** Stephen C. Gammie

**Affiliations:** Department of Integrative Biology, University of Wisconsin-Madison, Madison, USA

**Keywords:** Diseases of the nervous system, Genetics of the nervous system, Depression

## Abstract

Depression is a complex mental health disorder that is difficult to study. A wide range of animal models exist and for many of these data on large-scale gene expression patterns in the CNS are available. The goal of this study was to evaluate how well animal models match human depression by evaluating congruence and discordance of large-scale gene expression patterns in the CNS between almost 300 animal models and a portrait of human depression created from male and female datasets. Multiple approaches were used, including a hypergeometric based scoring system that rewards common gene expression patterns (e.g., up-up or down-down in both model and human depression), but penalizes opposing gene expression patterns. RRHO heat maps, Uniform Manifold Approximation Plot (UMAP), and machine learning were used to evaluate matching of models to depression. The top ranked model was a histone deacetylase (HDAC2) conditional knockout in forebrain neurons. Also highly ranked were various models for Alzheimer’s, including APPsa knock-in (2nd overall), APP knockout, and an APP/PS1 humanized double mutant. Other top models were the mitochondrial gene HTRA2 knockout (that is lethal in adulthood), a modified acetylcholinesterase, a Huntington’s disease model, and the CRTC1 knockout. Over 30 stress related models were evaluated and while some matched highly with depression, others did not. In most of the top models, a consistent dysregulation of MAP kinase pathway was identified and the genes NR4A1, BDNF, ARC, EGR2, and PDE7B were consistently downregulated as in humans with depression. Separate male and female portraits of depression were also evaluated to identify potential sex specific depression matches with models. Individual human depression datasets were also evaluated to allow for comparisons across the same brain regions. Heatmap, UMAP, and machine learning results supported the hypergeometric ranking findings. Together, this study provides new insights into how large-scale gene expression patterns may be similarly dysregulated in some animals models and humans with depression that may provide new avenues for understanding and treating depression.

## Introduction

Depression, including major depressive disorder (MDD), is a complex nervous system disorder in humans that is difficult to study and difficult to treat. A wide range of animal models exist for various neurological disorders^1–3^, including those for studying depression^4^, and for many of these data on large-scale gene expression patterns in the CNS are publicly available. For humans with depression, data on large-scale gene expression patterns in the CNS also exist that are derived from postmortem tissue from case MDD and control individuals^5–10^. Further, across multiple independent studies in humans, common depression dysregulation patterns have been identified, including dysregulation of MAP kinase signaling pathways^10,11^, suggesting that meaningful large-scale gene expression patterns exist with depression. Given large-scale gene expression data in animal models and humans with depression, it should be possible to identify the best matches of animal models to human depression to gain insights into how depression expression patterns can be triggered and what pathways are most commonly dysregulated^11^.

The goal of this study was to focus solely on large-scale gene expression patterns and compare and rank almost 300 animal models for congruence with human depression expression patterns. The models were mostly mice and rats and included those previously suggested to be models for studying depression along with other neurological disorder models, including those for Alzheimer’s or Parkinson’s disease, that can have comorbidity with depression^12,13^. The models were produced using a range of approaches, including transgenics, chemical/drug treatment, inbred strains, selection studies (e.g., for high anxiety), and environmental manipulation (e.g., chronic stress). Other models included natural variation such as female compared with male and postpartum compared with non-reproductive. All model large-scale gene expression data came from publicly available databases and were from nervous system tissue so comparison with CNS human data were consistent. Approaches for ranking included a hypergeometric based scoring system that rewards common gene expression patterns (e.g., up or down in both model and human depression), but penalizes opposing gene expression patterns. Overlapping genes between a given model and human depression were analyzed using enrichment tools. Further, machine learning that used human data for training and animal models for testing, Uniform Manifold Approximation Plot (UMAP)^14^ and Rank Rank Hypergometric Overlap (RRHO) heatmaps^15^ were used to evaluate matching of models with human depression. For the main focus of this study, human depression was represented by a single depression portrait that was recently created from 29 human depression datasets from males and females across multiple CNS regions and provides information on consistent patterns of dysregulation in depression across the CNS^11^. To help identify possible sex specific matches for human depression, separate analysis of a male and female portrait of depression with the models was also performed. Further, individual human datasets were also compared with the models. Given that a basis for drug repurposing is a reversal of gene expression patterns in a given disorder, the identification of animal models with high congruence with humans at the large-scale gene expression level could provide a new platform for evaluating the ability of new treatments for reversing depression expression patterns. The findings could also provide insights into how depression gene expression patterns can be triggered.

## Results

The top 20 animal models most congruent with human depression in terms of large-scale gene expression are shown in Table 1. The full ranking of all 289 models is provided in Supplementary Table 1. Overall, the top ranked model is the mouse transgenic with HDAC2 knockout (KO) in forebrain neurons. Ranking is based on inputs from hypergeometric analysis, but machine learning analysis also showed this as the top model (Supplementary Table 1). Ranked second was the Alzheimer's Disease (AD) mouse model with the APPsa knock in. The third ranked model was a KO of the mitochondrial gene, HTRA2. The fourth and fifth ranked models were also AD models, namely, the APP KO, and the APP/PS1 humanized transgenic. Seven of the top 20 most congruent models datasets with depression were related to AD pathways. Additional top 20 models included a Huntington's disorder model, an acetylcholinesterase (ACHE) variant model, three stress-related models, KOs of the Parkinson's related gene, LRRK2, KOs for the immune-related gene, STAT1, KOs of the DNA binding gene, INSM1, and KOs of the CREB1 coactivator gene, CRTC1 (Table 1). The ranking also allowed for identification of models most discordant with depression and among these was the treatment of sleep deprivation, suggesting this treatment could reverse depression gene expression patterns. When comparing all models against an alternate human portrait of depression created using metaVolcano, the hypergeometric scoring results were highly similar (Pearson correlation r = 0.822, pvalue < 0.0001 when comparing ranks; Supplementary Table 1).

**Table 1:** Top 20 models with highest congruence to human depression.

Rank	Model #	GEO #	Genotype or treatment	Sex	Source; age
1	m142	GSE93918	HDAC2 KO in forebrain^16^	Male	pfc; 8–20 weeks
2	m2	GSE25926	APPsa knock-in^29^	Male	pfc; 24–28 weeks
3	m9	GSE13033	HTRA2 KO^65^		Cortex; day 10–17
4	m1	GSE25926	APP knockout^29^	Male	pfc; 24–28 weeks
5	m144	GSE85162	APP/PS1	Male	Hippocampus; 8 months
6	m5	GSE31458	ACHE-R^34^	Both	pfc; 4 months
7	m56	GSE151807	chronic mild stress^66^	Male	Cerebral cortex; 4 weeks
8	m7	GSE3621	R6/1 HD^67^	Both	Brain hemisphere; 18 weeks
9	m8	GSE3621	R6/1 HD^67^	Both	Brain hemisphere; 27 weeks
10	m143	GSE85162	APP/PS1	Male	Frontal cortex; 8 months
11	m3	GSE25926	APLP2 knock-out^29^	Male	pfc; 24–28 weeks
12	m14	GSE33057	STAT1 KO^68^		Hippocampal neurons; e15-16
13	m241	GSE52584	LRRK2 KO^69^		Striatum; 4 months
14	m127	GSE81672	chronic stress; resilient^70^		Basolateral amygdala
15	m132	GSE90962	subchronic stress^71^	Female	Nucleus accumbens
16	m146	GSE85162	APP/PS1	Female	Hippocampus; 8 months
17	m145	GSE85162	APP/PS1	Female	Frontal cortex; 8 months
18	m262	GSE7707	MPTP treatment		Striatum
19	m57	GSE46139	INSM1 KO^72^		Pituitary; embryonic day 17
20	m147	GSE80633	CRTC1 KO^36^	Female	Cortex; 2 months

Ranking is based on the hypergeometric score that rewards matching in same direction (up-up and down-down) while penalizing opposite patterns. Full scoring details provided in Supplementary Table 1. Each of the top models was from mice.

pfc, prefrontal cortex; e15-16, embryonic days 15 and 16. References (if available) are shown under genotype.

**Figure 1:**
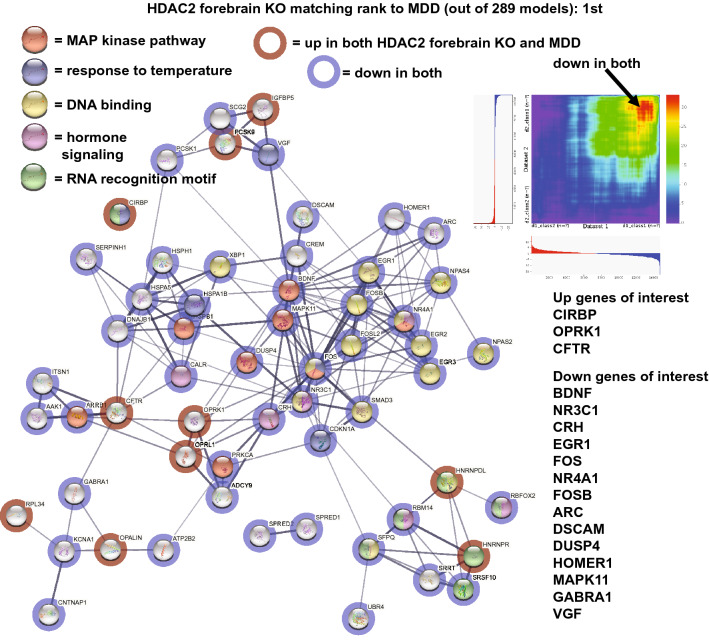
Congruent gene expression in HDAC2 forebrain KO mice and human depression. A subset of genes either upregulated (red circles) or downregulated (blue circles) in the CNS of both the HDAC2 forebrain KO and human depression are plotted in STRING^64^ that highlights gene–gene interactions (lines). Enrichment of matching genes included MAP kinase pathway (red), response to temperature (blue), DNA binding (yellow), hormone signaling (pink), and RNA recognition motif (green). Common up genes of interest were: CIRBP, OPRK1, and CFTR. Common down genes of interest included: BDNF, NR3C1, CRH, EGR1, FOS, NR4A1, FOSB, ARC, DUSP4, HOMER1, and VGF. An RRHO heatmap^15^ of the models with depression (X axis) is shown in upper right and high matching of down genes is highlighted by the arrow. The HDAC2 forebrain KO was ranked most congruent with human depression out of 289 models.

In order to evaluate how the HDAC2 forebrain KO matched human depression, a subset of highly connected congruent genes (up-up or down-down) were plotted in STRING (Fig. 1). As shown, upregulated genes included the cold-induced RNA binding protein (CIRBP), an opioid receptor, OPRK1, and the cystic fibrosis-related gene, CFTR. Notable downregulated genes matching depression were: BDNF, the glucocorticoid receptor, NR3C1, and activity related genes, EGR1, FOS, NR4A1, FOSB, and ARC. The neuropeptide, CRH, was also downregulated. Enriched pathways included: MAP kinase, response to temperature, DNA binding, and hormone signaling as shown in Fig. 1. An RRHO heatmap of the relation of the model with the depression portrait is shown in Fig. 1 and highlights the strong matching of down regulated genes in the two datasets.

**Figure 2:**
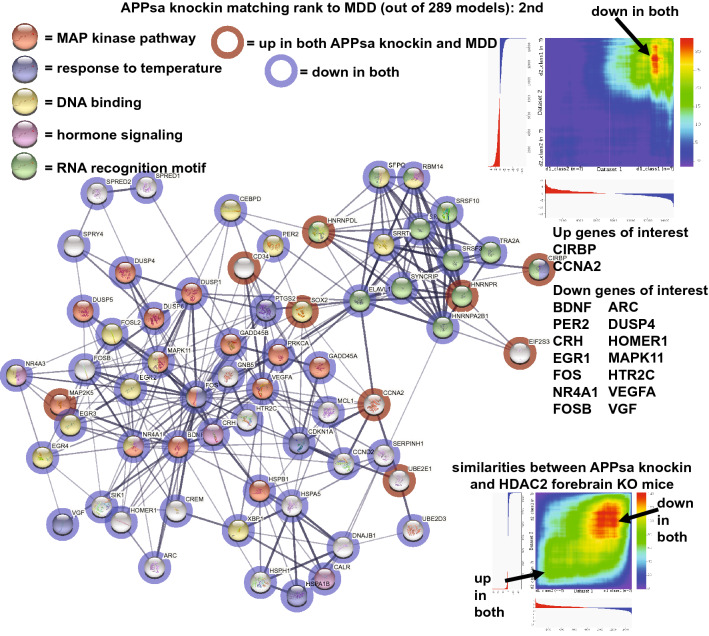
Congruent gene expression in APPsa knockin and human depression. A subset of genes either upregulated (red circles) or downregulated (blue circles) in the CNS of both the APPsa knockin and human depression are plotted in STRING^64^ that highlights gene–gene interactions (lines). Enrichment of matching genes included MAP kinase pathway (red), response to temperature (blue), DNA binding (yellow), hormone signaling (pink), and RNA recognition motif (green). RRHO heatmap of the two datasets (depression on X-axis) are shown in upper right and high matching of down regulated genes in both datasets is highlighted by the arrow. Common up genes of interest were: CIRBP and CCNA2. Common down genes of interest included: BDNF, PER2, CRH, EGR1, FOS, NR4A1, FOSB, ARC, DUSP4, HOMER1, HTR2C, and VGF. The APPsa knockin (X axis) and HDAC2 forebrain KO (Y axis) had similar expression profiles as shown in the RRHO heatmap (lower right) with common genes up and down shown by arrows. The APPsa knockin was the second highest ranked as congruent with human depression out of 289 models.

The APPsa knockin (second ranked) had a high matching of down regulated genes with depression (Fig. 2). Common down genes of interest included: BDNF, PER2, CRH, EGR1, FOS, NR4A1, FOSB, ARC, and VEGFA. Common up genes included CIRBP and CCNA2. Enrichment for MAP kinase pathway, response to temperature, DNA binding, and hormone signaling were also identified (Fig. 2). The RRHO heatmap (upper right of Fig. 2) highlights matching of downregulated genes in the model and human dataset. Analysis of the APPsa knockin with the HDAC forebrain KO revealed highly similar gene expression profiles as seen in the RRHO heatmap (lower right of Fig. 2).

**Figure 3:**
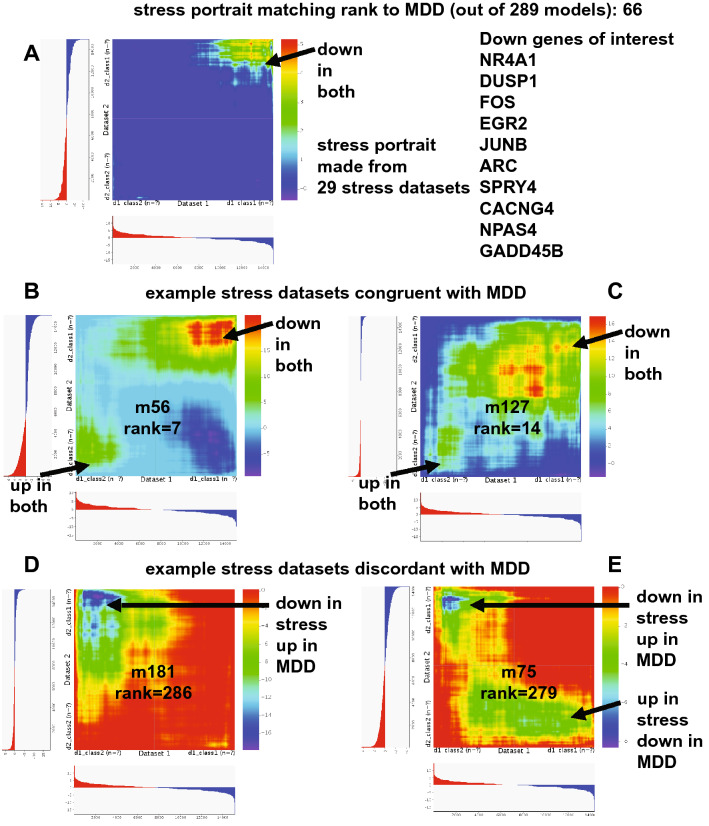
Stress datasets and variable congruence with human depression. A stress portrait combining 29 stress datasets was the 66th ranked model for congruence with human depression and an RRHO heatmap^15^ (depression is X-axis) highlights high matching of down regulated genes in both (**A**). Common down genes of interest include: NR4A1, DUSP1, FOS, EGR2, JUNB, ARC, SPRY4, CACNG4, NPAS4, and GADD45B. While some individual stress datasets were congruent with human depression (**B** and **C**), others were discordant (**D** and **E**) as shown in RRHO heatmaps (depression is X-axis in each). Number key: m56 = chronic mild stress; m127 = chronic stress in resilient group; m181 = chronic social defeat stress in susceptible group; m75 = social defeat stress in not susceptible group.

A subset of stress-related models ranked highly with human depression (three in the top twenty), but a number of others did not (Supplementary Table 1). Stress models ranged from social stress to mild chronic variable stress and there was no clear pattern of one type of stress better producing a match to human depression. A stress portrait derived from 29 stress datasets ranked as the 66th highest match to human depression and as shown in a heatmap in Fig. 3, there was mostly a matching of downregulated genes. Common down genes included: NR4A1, DUSP1, FOS, EGR2, JUNB, ARC, SPRY4, CACNG4, NPAS4, and GADD45B. Examples of heatmaps of individual stress datasets either matching or discordant with depression are shown in Fig. 3.

**Figure 4:**
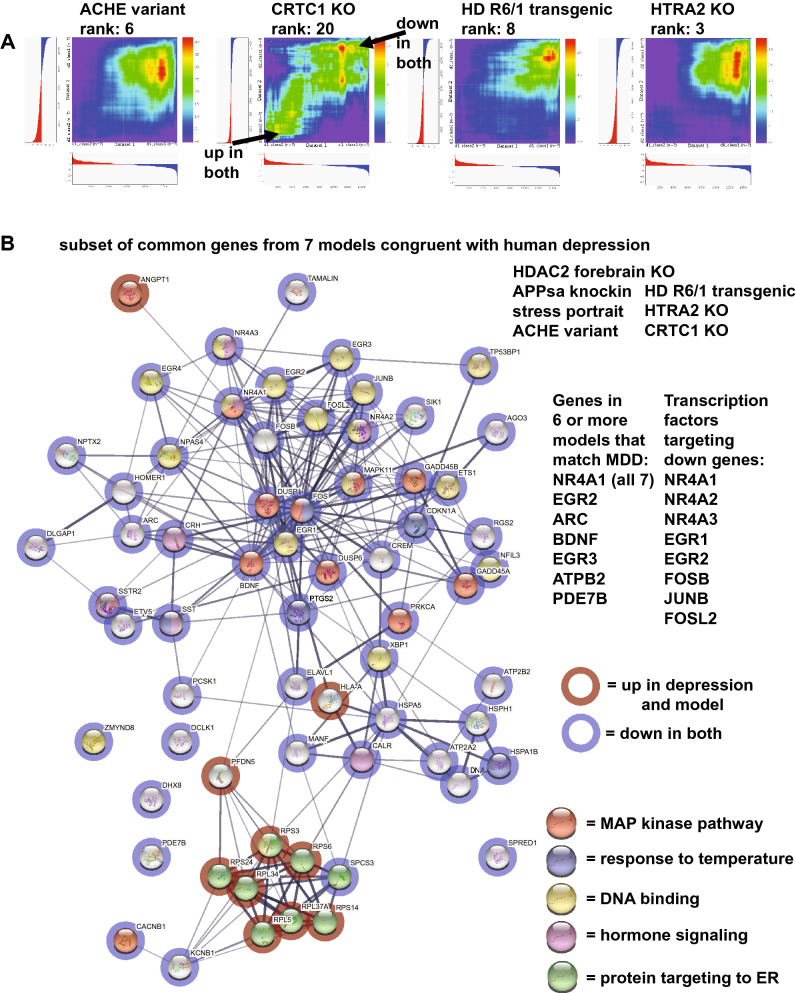
Common genes and pathway dysregulated in 7 different models that are congruent with human depression. RRHO heatmaps^15^ (human depression is X-axis) of additional congruent models with depression include: overexpression of an ACHE variant, a CRTC1 KO, an HD R6/1 transgenic, and a HTRA2 KO (**A**). The CRTC1 KO shows both strong matching to down and up regulated genes in depression (see arrows). For 7 models congruent with depression (HDAC2 forebrain KO, APPsa knockin, stress portrait, and the four highlighted in A, common genes were identified (in at least three of the models) and a subset are plotted in (**B**) using STRING interaction mapping^64^ (upregulation = red circles; downregulation = blue circles). Enrichment of matching genes included MAP kinase pathway (red), response to temperature (blue), DNA binding (yellow), hormone signaling (pink), and protein targeting to ER (green). NR4A1 was down in all 7 models and other genes in six of seven models were: EGR2, ARC, BDNF, EGR3, ATPB2, and PDE7B. When evaluating enrichment for transcriptional regulators of down common genes from the seven models, a subset of transcription factors were identified that themselves were downregulated, suggesting they could have a causal effect on producing a depressed pattern similar to depression and these included: NR4A1, NR4A2, NR4A3, EGR1, EGR2, FOSB, JUNB, and FOSL2.

RRHO heatmaps for the ACHE variant, CRTC1 KO, Huntington's disease (HD) R6/1 transgenic, and HTRA2 KO compared with human depression are shown in Fig. 4 While all exhibit matching to down regulated depression genes, CRTC1 also shows robust matching to up genes in depression. Seven models that matched depression from different approaches (HDAC2 forebrain KO, APPsa knockin, stress portrait, ACHE variant, HD R6/1 transgenic, CRTC1 KO, and HTRA2 KO) were analyzed and common up and down regulated genes with depression were identified (Fig. 4). NR4A1 was down in all seven models and depression. Genes down in 6 of 7 models were: EGR2, ARC, BDNF, EGR3, ATPB2, and PDE7B. When down genes matching depression (from at least 3 datasets) were analyzed in Enrichr, a connection to specific transcription factors was identified and of those, 8 genes were also among the downregulated genes, including NR4A1, NR4A2, NR4A3, EGR1, EGR2, FOSB, JUNB, and FOSL2, suggesting a possible causal link between a subset of transcription factors on depression patterns.

**Figure 5:**
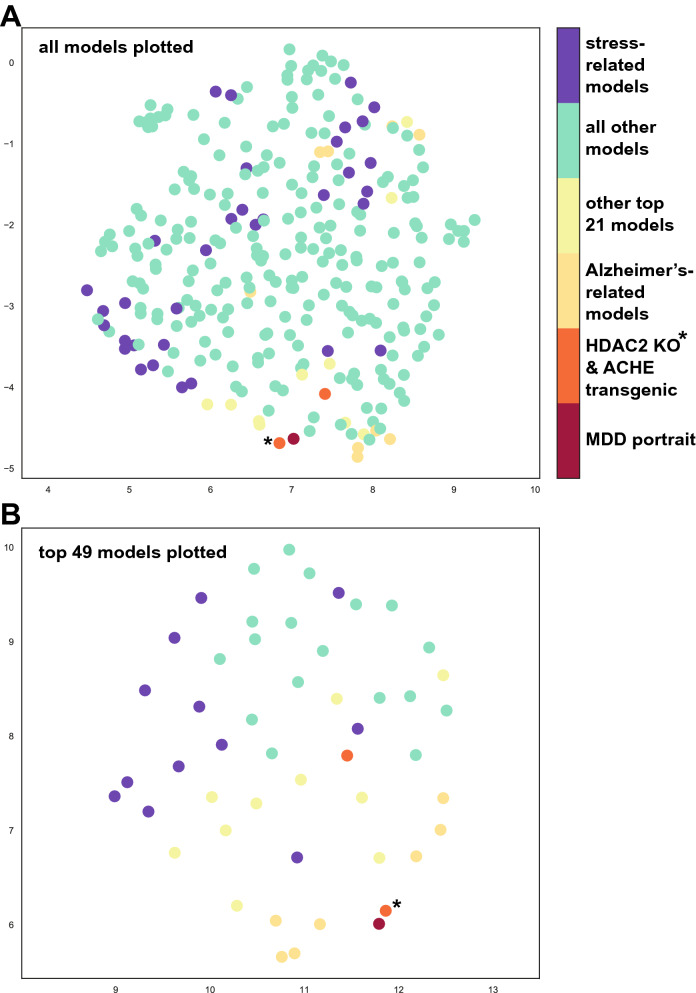
UMAP plotting of models with human depression. UMAP^14^ was used to incorporate data from complex landscape of multidimensional features (e.g., gene expression) and flatten those to two dimensions to identify similarities between datasets. All models and the human portrait of depression are plotted in (**A**), while only the top 49 models are plotted in (**B**). The closer distance (spatial proximity) of a model to human depression indicates a better match. The X‐ and Y‐axes are arbitrary embedding dimensions generated by UMAP. The top 2000 differentially expressed genes from the human depression portrait were used for plotting. As seen in (**A**) and (**B**), the HDAC2 forebrain KO (orange circle with asterisk) was consistently congruent with (close spatially to) the human depression (red circle). Most AD-related models (yellow circles) also were close spatially with the MDD portrait. As seen in A, some stress models (purple) were close to human depression spatially, but others were distant which is consistent with the ranking system. Other colored dots are: green = all other models; light yellow = other top 21 models; orange = ACHE transgenic.

Machine learning and UMAP were used as additional approaches to gain insights into how well models match the combined portrait of depression. Machine learning ranking results strongly matched those from the hypergeometric tests (Pearson correlation r = 0.76, pvalue < 10^−6^; Supplementary Table 1). In UMAP closeness spatially represents similarity and when all models are plotted, the HDAC forebrain KO is most closely aligned with the depression portrait (Fig. 5). Further, many AD-related models are plotted near the depression portrait. While some stress-related models are close to the depression portrait, a number are not and together the UMAP plotting matches the outcomes from the hypergeometric scoring and machine learning approaches.

When the male depression portrait was compared with each of the models, the overall ranking results were similar to the combined depression portrait: (Pearson correlation r = 0.80, pvalue < 10^−5^; Supplementary Table 1). Three of the top five matches for males were from APP/PS1 mutant mice. The HTRA2 KO and HDAC2 KO in forebrain neurons mouse were also in the top five. When the female depression portrait was compared with each of the models, the overall ranking results were also similar to the combined depression portrait (Pearson correlation r = 0.31, pvalue < 10^−5^; Supplementary Table 1). For female depression, chronic mild stress models were the top two models and the APPsa knock-in was third ranked. The HDAC2 KO in forebrains model was ranked sixth. Overall, the females matching scores to the models was less robust than for males, whereby the top score for females was 22.12, but for males it was 48.45. Overall, the male and female rankings were similar (Pearson correlation r = 0.24 pvalue < 0.000026), but there were instances of divergence in matches. For example, the top two male matches of APP/PS1 mutants showed only modest matching in females. For females, the 7th ranked model was short day versus long day, but this matching was not seen in males.

35 human depression datasets, including those used to create the portraits of depression, were compared with each of the animal models (Supplementary Table 2). Brain region information is provided for humans and animals so direct region to region comparisons can be made. As expected, the top congruent models with the portraits were found to be high matches in most of the individual datasets. No clear pattern of higher matching was found, though, when focusing only on specific brain regions in humans and animal models. For example, when examining dataset y15 (human male, cerebellum), the top matches were outside of cerebellum (cerebellum tissue from an APOD KO model was ranked 20th) (Supplementary Table 2). Any given human dataset and model dataset from the same or different region can be explored.

## Discussion

The goal of this study was to focus on large-scale gene expression in the CNS as the basis for evaluating congruence of animal models with that of human depression. The approach used allows for ranking of all models from most to least congruent with depression and for evaluation of genes that match or are the opposite of depression patterns. While the level of matching may be one level of interest, which specific genes or pathways match may be of greater interest.

The best match to the combined portrait of depression was the knockout mouse line where the histone deacetylase inhibitor, HDAC2, had been genetically removed from forebrain neurons (Table 1 and Fig. 1) and, interestingly, this line has been proposed as a model for schizophrenia^16^. The high congruence was largely due to common downregulated genes in the model and the human depression portrait. Down genes of interest included, BDNF, which is highly studied for its role in depression^17,18^. Another down gene was the glucocorticoid receptor, NR3C1, and this would be consistent with the idea of a lower energy state or low arousal as this receptor is activated during the transition from sleeping to waking^19^. The neuropeptide, CRH, was downregulated in the model and in depression. CRH is associated with anxiety and arousal, has a nonlinear effect on behavior when acting among multiple receptors, and is associated with psychiatric disorders, including depression^20–23^. A number of activity related genes, EGR1, FOS, NR4A1, FOSB, and ARC, were downregulated in the model and depression and this is consistent with the idea of the depressed brain being in a 'hypo' state^11^. The upregulation of the cold-induced RNA binding protein (CIRBP) along with decreased expression of multiple heat shock proteins suggests a possible decreased CNS temperature. CIRBP is of interest as it shows a circadian pattern of expression and affects expression of other genes^24^. Common gene upregulation included the opioid receptor, OPRK1, polymorphisms of which have been linked to mood disorders^25^ and the cystic fibrosis-related gene, CFTR which has been implicated in depression^26^. Enriched pathways included: MAP kinase, response to temperature, DNA binding, RNA recognition motif, and hormone signaling. Dysregulation of the MAP kinase pathway is linked to depression^10,27,28^.

The APPsa knockin that only has secreted APPs alpha^29^ ranked second with human depression and had a high matching of down regulated genes with depression (Fig. 2). Common down genes of interest included: BDNF, CRH, EGR1, FOS, NR4A1, FOSB, ARC, the circadian rhythm gene, PER2, that is linked to depression^30^, and VEGFA, with connections to depression^31^. Common up genes included CIRBP and CCNA2. Enrichment for MAP kinase pathway, response to temperature, DNA binding, RNA recognition motif, and hormone signaling were also identified (Fig. 2). Similarities of gene expression patterns were identified between the top two models as shown in the RRHO heatmap in Fig. 2 comparing the HDAC2 forebrain KO and the APPsa knockin. A possible connection between HDAC2 and Alzheimer's disease has been identified^32^ which could explain strong similarities of these two mouse lines at the gene expression level even though they were generated by manipulation of different gene targets. The link between AD-related mouse lines and depression was high with multiple AD models (e.g., humanized APP/PS1, APP KO, APLP2 KO) being among the top 20 matches and this finding is consistent with studies identifying high comorbidity of AD and depression^12,13^.

The application of stress to an animal model is often cited as a model for studying depression^33^. The best match was from chronic mild stress (Table 1) (ranked 7th) and three stress-related studies were in the top 20. The stress portrait built from 29 datasets matched positively with depression (ranked 66th) and included mostly common downregulated genes (Fig. 3). Among the down genes of interest were: NR4A1, DUSP1, FOS, EGR2, JUNB, ARC, SPRY4, CACNG4, NPAS4, and GADD45B. For male depression, the top ranked stress model was for chronic social defeat stress within resilient mice (ranked 11th) and for female depression the top two matches were chronic stress (Supplementary Table 1). However, for each of the three depression models a number of stress studies, including those with chronic stress or social defeat, had no relation to depression and some were highly discordant with genes in opposite patterns as depression (see Supplementary Table 1 and Fig. 3). Also, within studies, sometimes the group identified as resilient had better matches than those identified as susceptible and this could vary across strains and regions. The results of stress-related studies to depression expression profiles are complex. However, one interpretation is that given the stress portrait has similarities with depression expression and includes a core group of genes congruent with depression, there may be a fundamental link of stress to depression expression that can be found across studies. The results also suggest, though, that while stress can relate to depression gene expression, it is not necessarily a reliable cause of depression expression profiles and that genotype, stress type, and brain region play roles in outcomes. It should be noted that while this study used all publicly available stress datasets, the emergence of new stress-related datasets will improve our understanding of the connections of stress to depression expression.

Other models congruent with depression gene expression patterns included overexpression of acetylcholinesterase R variant that is less sensitive to neurotoxic MPTP treatment^34^. This finding is consistent with current studies evaluating links between acetylcholinesterase and depression^35^. The CREB-regulated transcription coactivator (CRTC1) KO ranked 20th and is noteworthy because it is one of the few top models that previously had been proposed as a model for studying depression^36^. Further, as seen in Fig. 4 and Supplementary Table 1, it was one of the few models that had solid matching of both up and down genes with depression. The Huntington's disease model, HD R6/1 transgenic, had a high match with depression and may reflect pathways that lead to comorbidity of HD and depression^37^. The KO of the mitochondrial gene, HTRA2, and its relation to depression may reflect the relationship of mitochondrial function with depression^38^, but given this KO is lethal in adulthood the utility of this model in studying depression may be limited.

Seven models that matched depression from different approaches (HDAC2 forebrain KO, APPsa knockin, stress portrait, ACHE variant, HD R6/1 transgenic, CRTC1 KO, and HTRA2 KO) were analyzed and common up and down regulated genes with depression were identified (Fig. 4). NR4A1 was down in all seven models. Genes down in 6 of 7 models were: EGR2, ARC, BDNF, EGR3, ATPB2, and PDE7B. When down genes matching depression (from at least 3 datasets) were analyzed in Enrichr, transcriptional regulation of these genes were found for 8 genes that themselves were downregulated, including NR4A1, NR4A2, NR4A3, EGR1, EGR2, FOSB, JUNB, and FOSL2. This finding is of interest as it suggests downregulation of a few transcriptional regulators may causally be triggering down regulation of a much larger number of depression-related genes. Thus, it is possible that targeting of these genes may have an oversized effect in treatments of depression.

One potentially interesting finding was the treatment of short day (versus long day) ranked 7th with women, but this matching was not seen in men. Although speculative, it is possible the findings could provide a link between depression in women and seasonal affective disorder^39^. Another potentially interesting result comes from analysis of the postpartum brain in two different mice strains. While the postpartum brain from the ICR-related strain^40–43^ does not match depression expression, the postpartum brain from the C57 strain brain^44^ does (ranked 47th). One idea is that for some individuals, maternity increases the risk of depression by pushing a subset of genes in a certain direction and in depression that same pattern exists. Thus, those maternity driven changes could be the basis of the vulnerability to postpartum depression. The result is consistent with the idea that there is a genetic component to vulnerability to postpartum depression^45^. Some of the common down genes in maternal C57 mice and depression were for the MAP kinase pathway and some of the common down genes included: FOS, EGR1, DUSP1, BDNF, NR4A1, NR3C1, the neuropeptide somatostatin (SST), and one of the its receptors, SSTR2. SST has been connected to depression^46^. None of these gene changes were found in ICR-related mothers suggesting that if the results are due to hormonal changes with motherhood, then they are not found in all strains. While this line of reasoning is speculative, it would be useful in future studies to identify in more detail whether or how gene expression changes in mothers are congruent or not with depression expression patterns and how that varies across genotype.

The ranking system also allowed for identification of models that were highly discordant with depression and have genes with opposing patterns. Of interest, the sleep deprivation approach in two different strains moved genes in opposite directions from depression and this is consistent with short term sleep deprivation as a treatment for depression^47^.

In this study there are a few technical considerations. The models come from a range of studies, including some where two genotypes (e.g., wild-type and KO) were injected with saline as part of a drug study. Here, only the saline injected animals of both groups would have been evaluated, but it is possible that the mild stress of injection helped to cause a divergence in gene expression. Most of the AD-related models were only examined in late adulthood so when differences appear at younger time points is not known. The HTRA2 KO it is lethal in adulthood and that may limit its usefulness for understanding depression. While all attempts were made to find relevant animal models for analysis, not all studies are publicly available, some relevant studies may have been missed, and it is likely that important new studies will be added. Given that the approach used highlighted the extent of matching, it is possible that some models were congruent with specific pathways dysregulated in human depression, but the overall scoring was modest. In future work, models could also be tested against specific pathways or gene sets dysregulated with depression. Further, possible models within the BxD recombinant inbred mice lines^48^ as well as the collaborative cross mice^49^ were not evaluated and it is possible that in future studies, a line with high congruence with depression will be identified. In this study a portrait of human depression was used that incorporated data from 29 human datasets from men and women across multiple brain regions and an advantage is that it provides a single consistent underlying signature of depression across the CNS for comparisons with the models^11^. The results from the separate analysis of the male and female portrait indicate both similarities and differences of matching to animal models. Additionally, the inclusion of results from individual human datasets to each of the models allows for comparisons across the same region to be made, but the limitation is that most human and animal models include only a few regions so comprehensive analysis that is region specific is limited. While the present study focused on common changes across the CNS in depression, future studies could focus more on data from specific brain regions (or cell types) from both humans and animal models.

The focus here was on large-scale gene expression and this approach provides a unique measure on how pathways dysregulated in depression can be elicited in various animal models. Drug repurposing includes the idea of reversing gene expression patterns^50^ and one possible future direction would be to use a highly congruent model with depression at the gene expression level as a platform for evaluating whether new treatments for depression can reverse that pattern in the CNS. Currently, testing of new depression treatments in animal models often focusses on behavioral changes, including endophenotypes^51–53^. A focus on reversing a dysregulated large-scale gene expression pattern that matches depression provides an alternative approach that is independent of a behavioral phenotype.

The findings using UMAP plotting (Fig. 5) were consistent with the hypergeometric and machine learning ranking system and the heatmaps from RRHO were useful in identifying the extent of matches across all genes. While ranking is useful, identification of the specific genes and pathways matching depression will likely have the most relevance in terms of any follow up studies using an animal model for studying depression. As noted above, it was of interest that 7 models created using different approaches had a convergence on a subset of pathways, including MAP kinase signaling, and genes that matched human depression (Fig. 4). This outcome is consistent with the idea that while the basis for depression at the SNP and GWAS level is complex^54,55^, there may be convergence of effects at the systems level. Understanding how different perturbations can lead to similar large-scale dysregulation could provide new insights into causes and treatments for depression.

## Methods

### Comparison of animal models' gene expression patterns with those in human depression

Large-scale gene expression analysis of animal models (microarray and RNA-seq) compared with controls were obtained from either the Gene Expression Omnibus (GEO)^56^ or GEO RNA-seq Experiments Interactive Navigator (GREIN)^57^. Only studies that evaluated expression in the CNS or nervous system related tissue were used so that comparisons with human CNS expression alterations with depression were relevant. Numerous brain regions were included in the model datasets, including cortex, hippocampus, retina, striatum, and cerebellum. The origin of tissue was evaluated as a factor against final ranking (ANOVA) and no effect was found. Depression was used as a key word to help identify potential depression models. Further, genes upregulated and downregulated in the human depression portrait^11^ were entered separately into Enrichr^58^ to identify additional potential models using the 'Gene Perturbations from GEO' feature. Models for Alzheimer's and Parkinson's were also included as both disorders have some overlap with depression^12,13^. Additional models included females compared with males in a given strain, one strain compared to another, and certain treatments, such stress exposure. Males and females were analyzed separately, whenever possible. In some cases, the providers of the datasets did not indicate sex and therefore the sex column was left blank. In some of these cases, the tissue was from younger animals when it is more difficult to accurately identify sex (e.g., early postnatal) and the authors did not attempt to identify the sex of the animal. In other cases, the authors indicated both sexes, but did not provide the information tag so that a separate analysis could be run. GSE numbers are provided for each study, so the reader can explore any of the studies in detail. In a few cases, datasets from similar categories were combined, such as for stress where different datasets used different stress approaches. A single portrait of stress was created that used 29 datasets to determine common effects of stress on the brain and this became an additional model tested. Other portraits were made for PINK1 knockout, females versus males (from multiple strains), and maternal versus non-maternal separately for two different strains. The approach for creating a portrait from multiple datasets is described in detail in^11^ and the key feature is that consistent gene expression changes in a given direction are highlighted. Prior to analysis, each dataset was formatted as described in detail in^11^, whereby gene symbols were updated to HUGO and pvalues were -log10 transformed and multiplied by the sign of direction change (positive = up; negative = down) so that a single number provides information on significance and direction of change. A full list of the 289 models tested is provided in Supplementary Table 1.

The human portrait of depression ("combined depression portrait") was used as the primary source for analysis as this dataset incorporates data from 29 studies on MDD across multiple brain regions, including cortex, striatum, nucleus accumbens, hippocampus, amygdala, and cerebellum in men and women^11^. A value of incorporating multiple datasets is that while each study attempted to control for demographics and other variables, the portrait would identify consistent changes and any unintended consequence of an uncontrolled variable in any one study would be minimized. Additionally, a male specific portrait and female specific portrait of depression were also analyzed^11^. Each animal model dataset was evaluated against human depression using a rank rank hypergeometric approach comparing the top 1000 upregulated and top 1000 downregulated genes from each dataset. Analysis was run in R^59^. The labeling system was the same as is used in the rank-rank hypergeometric publications^15^: A, upregulated in both groups; B, up in model, but down in depression; C, down in model, but up in depression; D, downregulated in both groups. The scoring systems is the same as in^11^. The output of A (up in both) and D (down in both) were added together and then the outputs of B (opposite directions) and C (opposite directions) were subtracted to provide a final score. A higher positive score indicates a better match of the model to depression because there is a high number of genes moving in the same direction, but a low number of genes moving in the opposite direction. The rationale for using the cutoff of 1000 is that it is likely to reflect meaningful biological alterations^11^ and using a specific number allows all datasets to be treated equally for analysis. The outputs of the scoring of all the models to the different portraits of depression are provided in Supplementary Table 1. When the original human depression portrait was created from 29 studies an alternate portrait was created^11^ using the same datasets and MetaVolcano^60^ that uses different metrics. All model datasets were also compared with the metaVolcano depression portrait and results are provided in Supplementary Table 1. A Pearson correlation was performed on the ranks from the two outputs.

RRHO^15^ was also used to provide a heat map of matching between any given dataset and the human depression portrait. It was also used to make comparisons between any two datasets. RRHO provides information genes both congruent (same direction) and discordant (opposite direction) and is useful as output from all genes is plotted.

Machine learning was used to rank rodent models with human depression using two training sets and two classifiers. The first training set used the same 29 formatted human depression datasets that were used to create the human depression model. Because the formatting already indicates direction of change in depression, each dataset was also reversed in sign to provide data for control. For the second training set the top genes up and down in depression in the depression portrait were shuffled randomly 20 times, but in each case the sign remained the same. For control training the signs were again reversed. The first training set used the top 2000 genes in the depression portrait and in the second set the number was reduced to 1636 to remove some genes in humans that were not well represented in the animal datasets. The Weka platform was used along with two different classifiers; LibLinear and Deep leaning 4j (Dl4j)^61^. In both cases probability estimates were used to create a ranking as the output included both a prediction of category (depression or control) as well as evidence for that prediction. Whether the training data and classifiers had the potential to make accurate predictions was tested using the standard cross validation (tenfold) approach. For the first training dataset, the LibLinear classifier had an accuracy of 100% and the Dl4j classifier had an accuracy of 100%. For the second training dataset, the LibLinear classifier had and accuracy of 100% and the Dl4j classifier had an accuracy of 96.5%. Together, outputs from two types of training and two classifiers provided four ranking outputs and these were averaged to a final machine learning prediction. The ranks from the machine learning scoring system are shown in Supplementary Table 1. Machine learning approaches were not run on the MetaVolcano portrait. Because machine leaning only used data from the combined portrait that combined male and female results, it was not possible to examine separately the role of sex with that training set. Separate machine learning approaches using male or female only data were not used.

UMAP analysis^14^ was used as alternative exploratory approach to gain insights into the congruence between models and human depression. UMAP incorporates data from a complex landscape of multidimensional features (e.g. genes) and flattens those to two dimensions to identify similarities or differences between datasets. The more closely aligned the datasets are, then the closer they are spatially plotted. Plotting was performed using the top 2000 genes from the human depression portrait.

### Analysis of congruent genes

Genes either up-up or down-down in two datasets were analyzed using multiple approaches. For enrichment, ToppCluster^62^, Enrichr^58^, and STRING^63^ were used. Genes were also entered into STRING to gain insights into interactions with one another. STRING is a protein–protein interaction tool and top interacting proteins (with a minimum interaction score of 0.40 between any two genes) were identified and replotted to highlight the most connected genes. The rationale for this analysis is that highly interactive genes could synergize to have an oversized effect.

### Analysis tools and datasets

All gene expression datasets are publicly available and the human depression portraits of depression are available from a prior publication^11^. All scripts for analysis and files used for analysis are available upon request.

## Supplementary Information

Supplementary Legends.

Supplementary Information 1.

Supplementary Information 2.

## Data Availability

All original datasets are publicly available datasets as indicated. Script and files used are available upon request. Any additional information can be received from the author by request.
