# Supplementary material for: Evaluation of animal model congruence to human depression based on large-scale gene expression patterns of the CNS
Source: Sci Rep. 2022 Jan 7;12:108. doi: 10.1038/s41598-021-04020-1 (PMC8741816; doi:10.1038/s41598-021-04020-1)
Supplement: Supplementary file 1 — Supplementary Legends. [file 41598_2021_4020_MOESM1_ESM.docx]

**Supplementary Data**

**Supplementary Table 1**

Scoring and ranking of depression portraits with each of the animal models based on congruence of large-scale gene expression in the CNS. Model ranking based on machine learning. Scoring and ranking of depression portrait made using metaVolcano with each animal model.

**Supplementary Table 2**

Comparisons of individual human datasets (male and female) from multiple brain regions with each of the animal models. Scoring is based on congruence of large-scale gene expression in the CNS using a hypergeometric approach.
